# Geometric mechanogenomics: engineering boundary conditions for deterministic cell fate control

**DOI:** 10.3389/fcell.2026.1922536

**Published:** 2026-08-20

**Authors:** Mohsen Taghizadeh, Ali Taghizadeh, Hye Sung Kim

**Affiliations:** 1 Institute of Tissue Regeneration Engineering (ITREN), Dankook University, Cheonan, Republic of Korea; 2 Mechanobiology Dental Medicine Research Center, Dankook University, Cheonan, Republic of Korea; 3 Department of Nanobiomedical Science and BK21 NBM Global Research Center for Regenerative Medicine, Dankook University, Cheonan, Republic of Korea

**Keywords:** genome organization, geometric mechanogenomics, mechanobiology, mechanogenomics, nuclear mechanics

## Abstract

In tissue development and regeneration, cellular behavior has traditionally been interpreted through biochemical signaling frameworks. However, cells exist within physically defined environments, where geometric boundary conditions – including confinement, curvature, anisotropy, and multicellular architecture – define the mechanical state space in which mechanical forces are generated, transmitted, and interpreted. Here, we introduce geometric mechanogenomics, a conceptual framework that positions geometry as an upstream spatial regulator linking tissue-scale boundary conditions to nuclear mechanics, chromatin organization, and genome regulation. We propose a boundary-to-nucleus axis through which geometric information is decoded by adhesion-mediated mechanotransduction, cytoskeletal force transmission, and nuclear mechanoregulation to regulate chromatin accessibility, epigenetic remodeling, and transcriptional programs. Rather than introducing new mechanotransduction pathways, this framework emphasizes that geometry spatially organizes conserved mechanotransductive machinery to generate context-dependent mechanogenomic outcomes. We further discuss how engineered geometries reduce morphogenetic stochasticity, coordinate multicellular organization, and establish mechanical memory that influences long-term cell fate. Finally, we highlight current challenges in establishing predictive geometry-to-genome relationships and discuss emerging opportunities enabled by spatial omics, artificial intelligence-assisted inverse design, and dynamic biomaterials for programmable mechanobiology, regenerative medicine, developmental biology, and disease modeling.

## 1 Introduction

The orchestration of tissue development and regeneration has traditionally been interpreted through biochemical frameworks, including morphogen gradients and transcriptional networks. Although these frameworks have provided fundamental insights into cell fate specification, they do not fully explain why genetically identical cells frequently adopt distinct phenotypes within the same biochemical milieu. Increasing evidence indicates that cellular behavior is also governed by the physical organization of the microenvironment. Cells exist within geometrically defined tissues, where boundary conditions – including confinement, curvature, anisotropy, adhesive topology, and multicellular architecture – govern how mechanical information is generated, transmitted, and interpreted (McBeath et al., 2004; Nelson et al., 2005; Théry et al., 2006; Jain and Vogel, 2018). Geometry should therefore be regarded as a fundamental physical regulator of cellular decision-making rather than merely a structural attribute of tissues.

Mechanical regulation is often discussed collectively, yet different physical cues encode distinct information. Matrix stiffness and viscoelasticity primarily determine the magnitude and temporal dissipation of mechanical resistance (Engler et al., 2006; Chaudhuri et al., 2016), whereas geometric boundary conditions specify where forces originate, along which spatial axes they propagate, and which cellular and nuclear structures experience mechanical loading. Although both engage conserved mechanotransductive machinery, stiffness primarily modulates the intensity of force transmission, whereas geometry organizes its spatial distribution. Geometry should therefore be viewed not as another mechanical variable but as an upstream boundary condition that determines how conserved mechanotransductive pathways are deployed.

Recent advances in bioengineering and mechanobiology have independently highlighted the importance of geometric regulation. Microfabrication, biomaterials engineering, and organoid technologies have demonstrated that engineered geometric constraints improve morphogenetic reproducibility by directing symmetry breaking, tissue patterning, and multicellular organization (Gjorevski et al., 2022; Mitrofanova et al., 2024; Kim et al., 2024; Maimets et al., 2026). In parallel, mechanobiology has revealed that mechanical forces regulate nuclear transport, chromatin organization, epigenetic remodeling, and transcriptional programs, giving rise to mechanogenomics (Jain et al., 2013; Roy et al., 2018; Swift et al., 2013; Walker et al., 2021). Despite their common physical basis, these fields have largely evolved independently. Engineering studies often end with tissue morphology or phenotypic outcomes, whereas mechanogenomic studies typically begin at the level of nuclear regulation, leaving unresolved how geometric information is transmitted across biological scales from tissue architecture to genome function.

**FIGURE 1:**
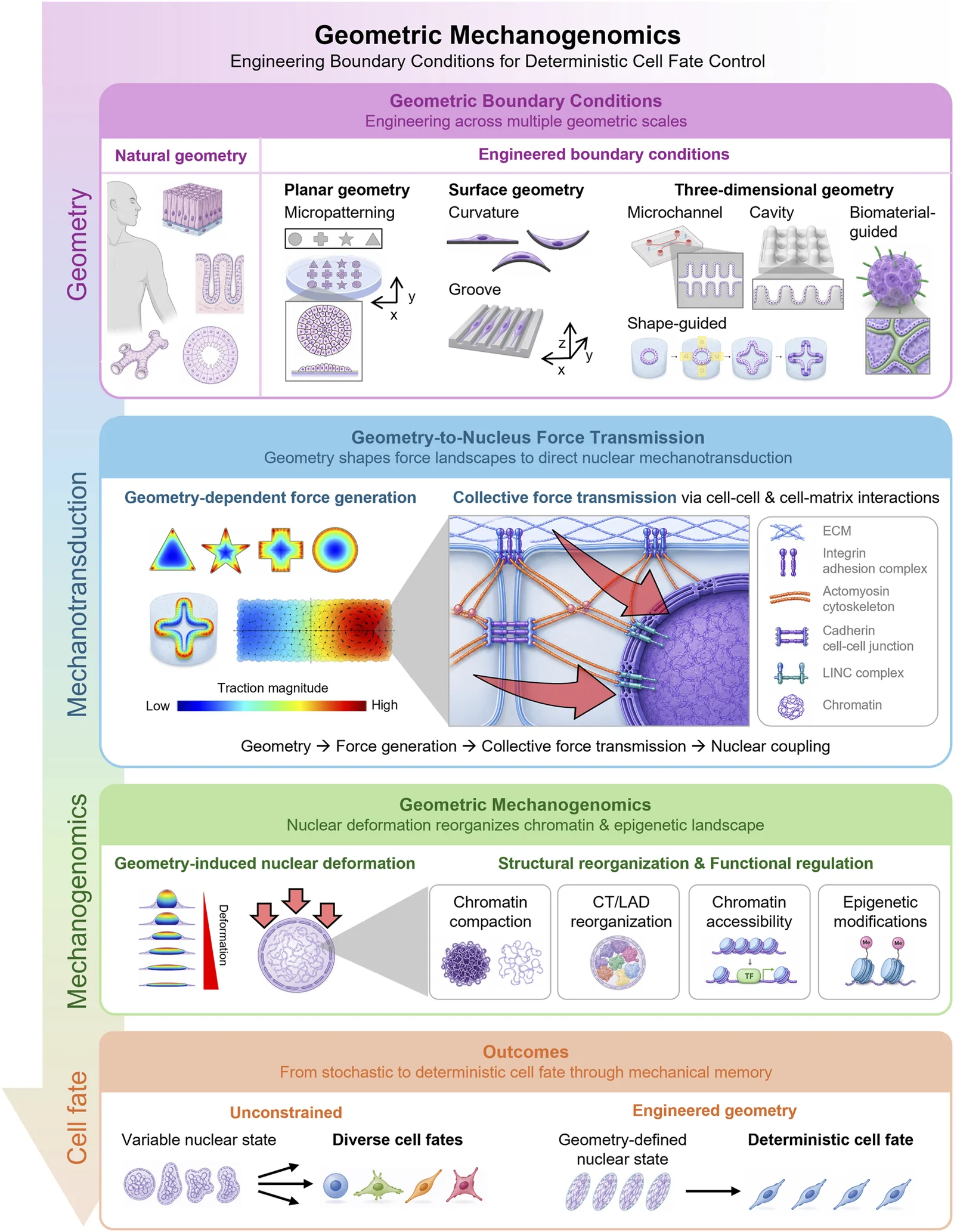
Geometric mechanogenomics: a multi-scale framework linking geometric boundary conditions to deterministic cell fate.

Here, we propose geometric mechanogenomics, a conceptual framework that positions geometric boundary conditions as programmable upstream regulators linking tissue architecture to genome regulation (Figure 1). Central to this framework is a boundary-to-nucleus axis, in which geometric constraints are translated into intracellular forces through adhesion-mediated mechanotransduction and subsequently reorganize nuclear architecture, chromatin accessibility, epigenetic states, and transcriptional programs. Rather than introducing new mechanotransductive pathways, geometric mechanogenomics proposes that geometry spatially organizes conserved mechanotransductive machinery to generate context-dependent mechanogenomic outcomes. By integrating advances in bioengineering, mechanobiology, and genome regulation, this framework establishes a conceptual foundation for the predictive engineering of cell fate through programmable physical boundary conditions.

Natural tissue architectures and engineered boundary conditions – including planar, surface, and three-dimensional geometries – define distinct mechanical state spaces by organizing adhesion topology, force generation, and collective force transmission. These geometry-dependent mechanical states are interpreted through conserved mechanotransductive machinery, including cell-matrix and cell-cell adhesions, the actomyosin cytoskeleton, and the LINC complex, to establish mechanically organized nuclear states. Geometry-induced nuclear deformation subsequently reorganizes chromatin architecture, chromosome territories, lamina-associated domains, chromatin accessibility, and epigenetic landscapes, collectively forming the mechanogenomic layer. The integration of these processes stabilizes nuclear organization and mechanical memory, biasing cell-state transitions from stochastic self-organization toward deterministic cell fate through programmable geometric boundary conditions.

## 2 Geometric control of cell fate: a multi-scale framework

### 2.1 Geometry as a boundary condition: from stochasticity to reproducible morphogenesis

A defining feature of embryonic development is its reproducibility despite the stochasticity of molecular interactions. Although gene expression, signaling dynamics, and cell fate decisions vary at the single-cell level, developing tissues consistently generate ordered architectures. By contrast, engineered organoids frequently exhibit substantial heterogeneity in size, morphology, and tissue organization, suggesting that biochemical self-organization alone is insufficient to ensure reproducible morphogenesis. One reason is that *in vitro* systems often lack the geometric boundary conditions that constrain and organize cellular behavior during development.

In native tissues, cells develop within spatially defined environments characterized by confinement, curvature, anisotropy, epithelial boundaries, and multicellular packing. These features restrict where cells proliferate, migrate, establish adhesions, and generate forces. Tissue geometry therefore does more than provide structural support; it organizes collective behavior by defining the physical context in which developmental programs unfold. Reproducing these boundary conditions has consequently emerged as a powerful strategy for reducing morphogenetic variability and improving tissue reproducibility.

Advances in biomaterials, microfabrication, and organoid engineering demonstrate that precisely engineered geometries can shift stochastic developmental processes toward more deterministic outcomes. Early micropatterning studies showed that colony geometry determines mammary branching sites and spatially patterns embryonic stem cell differentiation (Nelson et al., 2006; Warmflash et al., 2014). More recently, engineered crypt architectures, microfabricated cavities, permeable membranes, and scaffold-guided organoid systems have shown that predefined geometric constraints reproducibly regulate symmetry breaking, tissue patterning, multicellular organization, and organoid maturation (Lancaster et al., 2017; Gjorevski et al., 2022; Ritzau-Reid et al., 2023; Kim et al., 2024; Lorenzo-Martín et al., 2025; Maimets et al., 2026). Geometry is therefore not merely permissive for development but actively constrains the developmental landscape.

Importantly, geometry is not a direct source of biological information or force. Instead, it defines the mechanical state space within which forces emerge. Even in mechanically homogeneous environments, heterogeneous traction patterns arise through differences in cell spreading, polarity, focal adhesion assembly, actomyosin contractility, and cell-cell interactions. Geometry prescribes where cells adhere, where stresses concentrate, how collective forces propagate, and which regions of the cytoskeleton and nucleus experience mechanical loading. It therefore functions as an upstream organizational framework rather than an independent mechanical stimulus.

This role distinguishes geometric boundary conditions from bulk matrix mechanics (Table 1). Matrix stiffness and viscoelasticity regulate the magnitude and temporal dissipation of force transmission within an established physical environment (Engler et al., 2006; Chaudhuri et al., 2016), whereas geometry organizes that environment by prescribing its spatial constraints. Cells cultured on substrates with identical stiffness may therefore establish distinct mechanical states because their adhesive geometry, confinement, curvature, or multicellular organization differs. Adhesive micropatterning studies have shown that cell shape, aspect ratio, and confinement reorganize focal adhesions, stress fibers, RhoA/ROCK signaling, and lineage specification without altering matrix chemistry or stiffness (McBeath et al., 2004; Théry et al., 2006; Kilian et al., 2010; Callens et al., 2020). Likewise, YAP/TAZ activation depends strongly on cytoskeletal organization and cell spreading rather than simply on the absolute area of cell-matrix contact (Dupont et al., 2011).

**TABLE 1:** Conceptual distinction between bulk matrix mechanics and geometric boundary conditions. Bulk matrix mechanical properties primarily modulate the magnitude and persistence of force transmission, whereas geometric boundary conditions define the mechanical state space by organizing where, how, and between which cellular structures forces are generated and propagated.

Conceptual comparison		
Feature	Bulk matrix mechanical properties	Geometric boundary conditions
Encoded physical information	Material resistance and temporal mechanical behavior	Spatial organization of the cellular microenvironment
Primary regulatory role	Modulates the magnitude and persistence of force transmission	Defines the mechanical state space by organizing where, how, and between which cellular structures forces are generated and propagated
Representative parameters	- Elastic stiffness - Viscoelasticity - Stress relaxation	- Confinement - Curvature - Adhesive geometry - Aspect ratio - Multicellular packing - 3D architecture
Engineering question	How much force is transmitted?	Where, how, and along which path are forces transmitted?
Overall concept	Controls mechanical resistance	Acts as an upstream spatial organizer of mechanotransduction and mechanogenomic regulation

Representative geometric boundary cues				
Geometric cue	Physical information	Primary organizational role	Mechanotransductive organization	Representative evidence
Adhesive area/spreading	Available spreading area	Defines where adhesion and tension develop	RhoA–ROCK–myosin II; YAP/TAZ	McBeath et al., 2004; Dupont et al., 2011
Shape/aspect ratio	Boundary anisotropy	Organizes cytoskeletal anisotropy and force orientation	Stress fibers; focal adhesions	Théry et al., 2006; Kilian et al., 2010; Jain et al., 2013
Multicellular topology	Cell–cell connectivity	Establishes collective force transmission	Cadherin–α-catenin–vinculin	Nelson et al., 2005; Mertz et al., 2013
Confinement	Space relative to cell/nucleus	Couples cell and nuclear dimensions	LINC complex; nuclear deformation	Roy et al., 2018; Lomakin et al., 2020
Curvature	Local bending constraints	Redistributes mechanical loading	Cytoskeletal alignment	Callens et al., 2020
3D architecture/crypt geometry	Boundary architecture and tissue organization	Creates regional mechanical niches	Spatial YAP/Notch signaling	Gjorevski et al., 2022; Maimets et al., 2026

The upper section summarizes the conceptual distinction, while the lower section provides representative geometric boundary cues and supporting evidence.

Collectively, geometric boundary conditions define the mechanical state space within which conserved mechanotransductive pathways operate, thereby biasing developmental trajectories toward reproducible outcomes. This upstream organizational role provides the conceptual basis for the boundary-to-nucleus axis described below.

### 2.2 The boundary-to-nucleus axis: geometry-defined mechanotransduction

The translation of geometric boundary conditions into biological information requires a continuous physical pathway through which spatially organized mechanical cues are transmitted from the extracellular environment to the nucleus. We refer to this hierarchical process as the boundary-to-nucleus axis, a geometry-defined mechanotransduction framework in which boundary conditions organize how forces are distributed, propagated, and ultimately interpreted by cells. Rather than constituting an independent signaling pathway, this axis describes the sequential conversion of geometric information into mechanically organized nuclear states.

Because geometric boundary conditions cannot directly regulate nuclear function, cells first interpret geometric information through their interface with the surrounding microenvironment. Geometric constraints – including confinement, curvature, anisotropy, and multicellular architecture – determine where adhesive contacts form and mature, establishing distinct patterns of integrin engagement, focal adhesion assembly, and intracellular tension generation. In this way, geometry defines the mechanical landscape before downstream mechanotransductive signaling is initiated (Hu and Bao, 2024; Mertz et al., 2013).

These geometry-defined adhesion patterns subsequently organize the actomyosin cytoskeleton into anisotropic networks that transmit forces toward the nucleus. Mechanical continuity is maintained through integrin-mediated adhesions, the actomyosin cytoskeleton, and the linker of nucleoskeleton and cytoskeleton (LINC) complex, which physically couples cytoskeletal tension to the nuclear envelope (Wang et al., 2009; Kalukula et al., 2022; Walker et al., 2021). Although integrins, focal adhesion kinase (FAK), RhoA/ROCK signaling, and actomyosin contractility constitute conserved components of mechanotransduction (del Rio et al., 2009; Grashoff et al., 2010; Elosegui-Artola et al., 2016), their spatial engagement is dictated by geometric boundary conditions (Théry et al., 2006; Yonemura et al., 2010). Geometry therefore does not introduce distinct mechanotransductive machinery but spatially organizes the activation of conserved pathways.

As mechanically organized forces converge on the nucleus, the nucleus functions as an active mechanosensory organelle rather than a passive recipient of force (Lomakin et al., 2020; Venturini et al., 2020). Geometric confinement, stretching, compression, and anisotropic loading deform the nucleus, altering nuclear mechanics and transport dynamics. Nuclear envelope deformation regulates nuclear pore permeability and the import of mechanosensitive transcriptional regulators such as YAP/TAZ (Dupont et al., 2011; Elosegui-Artola et al., 2017), while force transmission through the nuclear lamina reorganizes lamin architecture and chromatin tethering, influencing higher-order chromatin organization and genome accessibility (Nava et al., 2020).

These mechanically organized nuclear states do not themselves constitute mechanogenomic regulation but establish the physical conditions that enable subsequent changes in chromatin accessibility, transcription factor dynamics, epigenetic remodeling, and transcriptional programs. Collectively, the boundary-to-nucleus axis represents the first regulatory layer of geometric mechanogenomics, converting spatially encoded boundary conditions into mechanically organized nuclear architecture that becomes competent for genome-level regulation.

### 2.3 Geometric mechanogenomics: genome reconfiguration through boundary constraints

The structural and functional reconfiguration of the genome represents the ultimate downstream consequence of the boundary-to-nucleus axis. We define this geometry-driven regulatory framework as geometric mechanogenomics, a conceptual extension of mechanogenomics that positions geometric boundary conditions as programmable upstream regulators of genome function. Rather than focusing solely on force-responsive molecules, geometric mechanogenomics emphasizes how spatially organized boundary conditions reorganize nuclear architecture, chromatin organization, and transcriptional competence to establish predictable cell-state transitions.

Mechanically organized nuclear states provide the physical foundation for genome regulation (Uhler and Shivashankar, 2017). Sustained geometric confinement, anisotropic loading, and nuclear deformation impose persistent mechanical stresses that reorganize higher-order chromatin architecture (Jain et al., 2013; Lomakin et al., 2020). Emerging evidence indicates that geometric constraints influence chromosome territories, lamin-associated chromatin interactions, and the balance between heterochromatin and euchromatin, thereby reshaping three-dimensional genome organization (Nava et al., 2020). These structural changes alter chromatin accessibility and establish transcriptional landscapes that bias lineage-specific gene expression and developmental programs (Walker et al., 2021; Mitra et al., 2017).

These effects extend beyond transient mechanotransductive signaling. Mechanical inputs generated by geometric boundary conditions can induce stable epigenetic remodeling through changes in chromatin accessibility, histone modification, DNA methylation, and nuclear organization (Yang et al., 2014; Le et al., 2016; Maurer and Lammerding, 2019). Consequently, the genome serves not only as a molecular readout of mechanical stimulation but also as a repository of mechanical memory, in which previous physical experiences become persistently encoded within chromatin architecture and epigenetic landscapes.

A major implication of geometric mechanogenomics is its potential for mechanical reprogramming. Precisely engineered geometric microenvironments can reverse age-associated nuclear abnormalities, restore lamin organization, reorganize lamin-associated chromatin domains, and suppress senescence-associated phenotypes without direct genetic manipulation (Stephens et al., 2017; Roy et al., 2020). Similar principles are emerging in stem cell biology and regenerative medicine, where engineered boundary conditions enhance lineage specification, preserve developmental competence, and improve tissue maturation by reorganizing genome architecture rather than simply activating individual signaling pathways.

Collectively, these findings establish geometric mechanogenomics as a hierarchical framework linking programmable boundary conditions to genome organization and cell fate. Rather than directly manipulating genes, engineered geometric constraints reorganize genome architecture to establish predictable transcriptional states and developmental trajectories, providing a conceptual foundation for engineering cell identity through programmable physical boundary conditions.

## 3 Discussion: toward predictive and programmable mechanobiology

### 3.1 Competing paradigms: plasticity versus programmed stability

A central challenge in geometric mechanogenomics is reconciling two long-standing perspectives on cell fate regulation. Engineering-oriented studies emphasize that cells exhibit remarkable mechanical plasticity and can be redirected through manipulation of their physical microenvironment (Engler et al., 2006; Kilian et al., 2010). In contrast, developmental biology argues that robust gene regulatory networks constrain developmental trajectories and confer resistance to environmental perturbation (Davidson et al., 2010). Rather than representing opposing paradigms, these views likely describe complementary aspects of the same biological system.

From the perspective of geometric mechanogenomics, the key question is not whether cell fate is mechanically plastic or genetically predetermined, but how geometric boundary conditions reshape the landscape within which genetically permissible cell states are selected. Rather than overriding intrinsic developmental programs, geometry biases developmental trajectories by reorganizing the mechanical and genomic contexts in which those programs operate. This perspective shifts the focus from deterministic genetic instruction to probabilistic regulation of cell-state transitions through spatially organized physical environments.

A related challenge concerns the persistence of mechanically induced cellular states. Geometric confinement can induce nuclear deformation, cytoskeletal remodeling, and chromatin reorganization, but whether these responses represent transient adaptation, reversible priming, or stable lineage commitment likely depends on mechanical dose, exposure duration, developmental stage, and biochemical context. Although the concept of mechanical memory describes the retention of mechanically encoded cellular states (Yang et al., 2014), its duration, reversibility, and molecular basis remain unclear. Addressing these questions will require long-term lineage tracing, temporal release experiments, targeted perturbation of adhesion-cytoskeletal and LINC-mediated coupling, and integrated chromatin and transcriptomic analyses following removal of geometric constraints. Resolving when geometry induces reversible adaptation versus stable commitment will ultimately define the scope of geometric mechanogenomics.

### 3.2 The missing multi-scale link: from geometry to genome

Despite rapid progress, a fundamental gap remains in linking engineered geometric inputs to genome-scale outputs. Bioengineered platforms precisely control confinement, curvature, anisotropy, and multicellular architecture, yet many studies still rely primarily on morphological or marker-based endpoints. Conversely, genomic and epigenomic analyses provide high-resolution molecular information but rarely incorporate well-defined geometric boundary conditions. Consequently, the field still lacks quantitative frameworks capable of predicting how defined geometric parameters are translated into specific mechanical states, chromatin architectures, and transcriptional programs.

Bridging this gap will require integrated multi-scale platforms that simultaneously control geometry, quantify force transmission, and resolve nuclear and genomic organization at single-cell resolution. Such systems should combine engineered microenvironments with traction-force microscopy or force-inference methods, live imaging of nuclear mechanics and mechanosensitive transcription factors, targeted perturbation of adhesion complexes, cytoskeletal regulators, the LINC complex, lamins, and chromatin remodeling machinery, together with spatially resolved transcriptomic and epigenomic analyses. Integrating these approaches will establish causal relationships between boundary geometry, mechanical state, nuclear architecture, and genome regulation.

Ultimately, the goal extends beyond generating increasingly complex datasets. The field requires predictive quantitative models that translate geometric descriptors into mechanical states and genome organization across biological scales. Achieving this capability would transform geometric mechanogenomics from a descriptive framework into a predictive engineering theory for controlling cell-state regulation.

### 3.3 Outlook: toward predictive engineering of cell fate

The framework of geometric mechanogenomics extends beyond a conceptual reinterpretation of mechanobiology and suggests new strategies for engineering cell fate through programmable physical boundary conditions. Rather than treating geometry as a passive scaffold or experimental constraint, this perspective positions boundary conditions as design parameters that can be systematically engineered to regulate genome organization and cellular function.

This shift has broad implications across developmental biology, regenerative medicine, disease modeling, and biomaterials engineering. Advances in microfabrication, biomaterials, organoids, and bioprinting increasingly enable precise control over geometric boundary conditions, creating opportunities to engineer tissue development through physical design rather than biochemical optimization alone. Such approaches may improve developmental reproducibility, enhance tissue maturation, and increase the robustness of engineered biological systems.

Future progress will require integrating quantitative engineering, mechanobiology, and genome biology into unified predictive frameworks. Combining programmable geometric microenvironments with multi-scale mechanical measurements, spatial omics, genome-wide analyses, and artificial intelligence-assisted inverse design will enable the establishment of causal design rules linking boundary geometry to nuclear organization, genome regulation, and cell fate (Bray et al., 2016; Caicedo et al., 2017; Ching et al., 2018). Ultimately, geometric mechanogenomics provides a conceptual foundation for transforming tissue engineering from empirical optimization toward predictive, geometry-guided control of cellular identity and function.
